# The new insights into autophagy in thyroid cancer progression

**DOI:** 10.1186/s12967-023-04265-6

**Published:** 2023-06-24

**Authors:** Yu-Bo Shi, Shu-Yuan Chen, Ren-Bin Liu

**Affiliations:** Department of Thyroid and Breast Surgery, The Third Affiliated Hospital of Sun Yat-Sen University, Guangzhou, China

**Keywords:** Thyroid cancer, Autophagy, Cancer progression, Autophagy inhibitor, Targeted therapy

## Abstract

In recent decades, the incidence of thyroid cancer keeps growing at a shocking rate, which has aroused increasing concerns worldwide. Autophagy is a fundamental and ubiquitous biological event conserved in mammals including humans. Basically, autophagy is a catabolic process that cellular components including small molecules and damaged organelles are degraded for recycle to meet the energy needs, especially under the extreme conditions. The dysregulated autophagy has indicated to be involved in thyroid cancer progression. The enhancement of autophagy can lead to autophagic cell death during the degradation while the produced energies can be utilized by the rest of the cancerous tissue, thus this influence could be bidirectional, which plays either a tumor-suppressive or oncogenic role. Accordingly, autophagy can be suppressed by therapeutic agents and is thus regarded as a drug target for thyroid cancer treatments. In the present review, a brief description of autophagy and roles of autophagy in tumor context are given. We have addressed summary of the mechanisms and functions of autophagy in thyroid cancer. Some potential autophagy-targeted treatments are also summarized. The aim of the review is linking autophagy to thyroid cancer, so as to develop novel approaches to better control cancer progression.

## Background

Despite the fact that thyroid cancer (TC) accounts for 3% of new cancer cases in both males and females, it is the fifth common cancer types (4.9%) in female population, after breast, colorectum, lung and cervix uteri cancer [1]. And the data have been increasing over the years globally [2]. TC is either originated from follicular cells or parafollicular cells (C cells). Based on differentiation profiles, TC is overall divided into well-differentiated TC, poorly-differentiated TC and anaplastic TC (ATC). In differentiated TC, which accounts for 90–95% of all clinical cases and is able to take iodine, it is more subtly divided into papillary thyroid cancer (PTC), follicular thyroid cancer (FTC) and a less common one, the Hurthle cell TC [2, 3]. PTC carries the best prognosis as it barely metastasizes, if any, it is the surrounding cervical lymph nodes, whereas the FTC, Hurthle cell TC and poorly-differentiated TC have tendency of lung or bone metastasis via bloodstream. ATC is totally undifferentiated, so its histological origin is undefined. As ATC grows rapidly, it has a poor prognosis [3, 4]. In contrast, the C cells-derived cancer is called medullary TC, which accounts for 2–4% of all cases and easy to have cervical lymph nodes metastasis [5]. Surgical removal is recommended for the primary lesions. Radioactive iodine (RAI) uptake is one of the most powerful and effective methods to treat metastasized differentiated TC, known as the internal radiotherapy [6]. Up to now, multiple targeted drugs like cabozantinib, vandetanib, sorafenib and lenvatinib have also been introduced into clinical trials or uses for advanced TC [7]. Owing to the multimodality therapies, the mortality rate of TC is declining over the years, in contrast to the rising incidence [8].

Although in most of the cases TC is not lethal, its high incidence cannot be overlooked. Thus, it is inevitable to unveil the mechanisms of tumorigenesis. Autophagy is widely conserved from yeast to humans, by which the damaged cellular components or inactivated molecules are recycled for new synthesis and reenter the metabolic processes. Autophagy is of high concern because it has crosstalk with cancers, ageing, neurodegenerative disorders, etc. In this review, we will mainly concentrate on how autophagy regulates thyroid tumorigenesis and how to manage this tumor via this approach.

## The overview of autophagy

Autophagy, together with ubiquitination, is essential for substance degradation [9]. Autophagy is a housekeeping process at a basal level and can be enhanced under unfavorable situations, such as hypoxia, starvation, stress and toxicity [10]. Autophagy classically contains three formats, namely, macroautophagy, microautophagy and chaperone-mediated autophagy. The macroautophagy is the most common one and is regarded as autophagy hereafter [11]. Autophagy is a self-protective action to maintain homeostasis, during which the misfolded or useless proteins are broken down in the lysosomal lumen and recycled for another biosynthesis. The autophagic process is kept in a subtle balance, neither too strong nor too weak. The enhancement of autophagy perhaps destroys normal cellular structure and affects physiological metabolism. Oppositely, if this event is deficient, many toxic or useless substances may be stored in cytosol, leading to pathological alterations, such as Alzheimer’s disease [12].

The cascades of autophagic processes can be generalized to several steps. In the beginning, autophagy initiation is induced intrinsically or extrinsically by some stimuli like starvation or oxidative stress. Structurally, the autophagic cascades begin with the formation of phagophore (vesicle nucleation) at several phagophore assembly sites in cytosol, after receiving external signals [11, 13]. Phagophore, or the isolation membrane, is remarked as a curve, bowl-like, double-layer membrane structure, which elongates, expands and surrounds the degraded targets such as damaged proteins, organelles and lipid droplets. Once the membrane ends fusion, the formation of autophagosome is accomplished [14, 15]. The mature autophagosome is further transported to the lysosome and fuses with it through the outer membrane, the new structure is thus called autolysosome. The cargos are ultimately degraded and recycled in the structure by lipases, proteases, nucleases, glycosylases and etc. [11, 15].

**Fig. 1:**
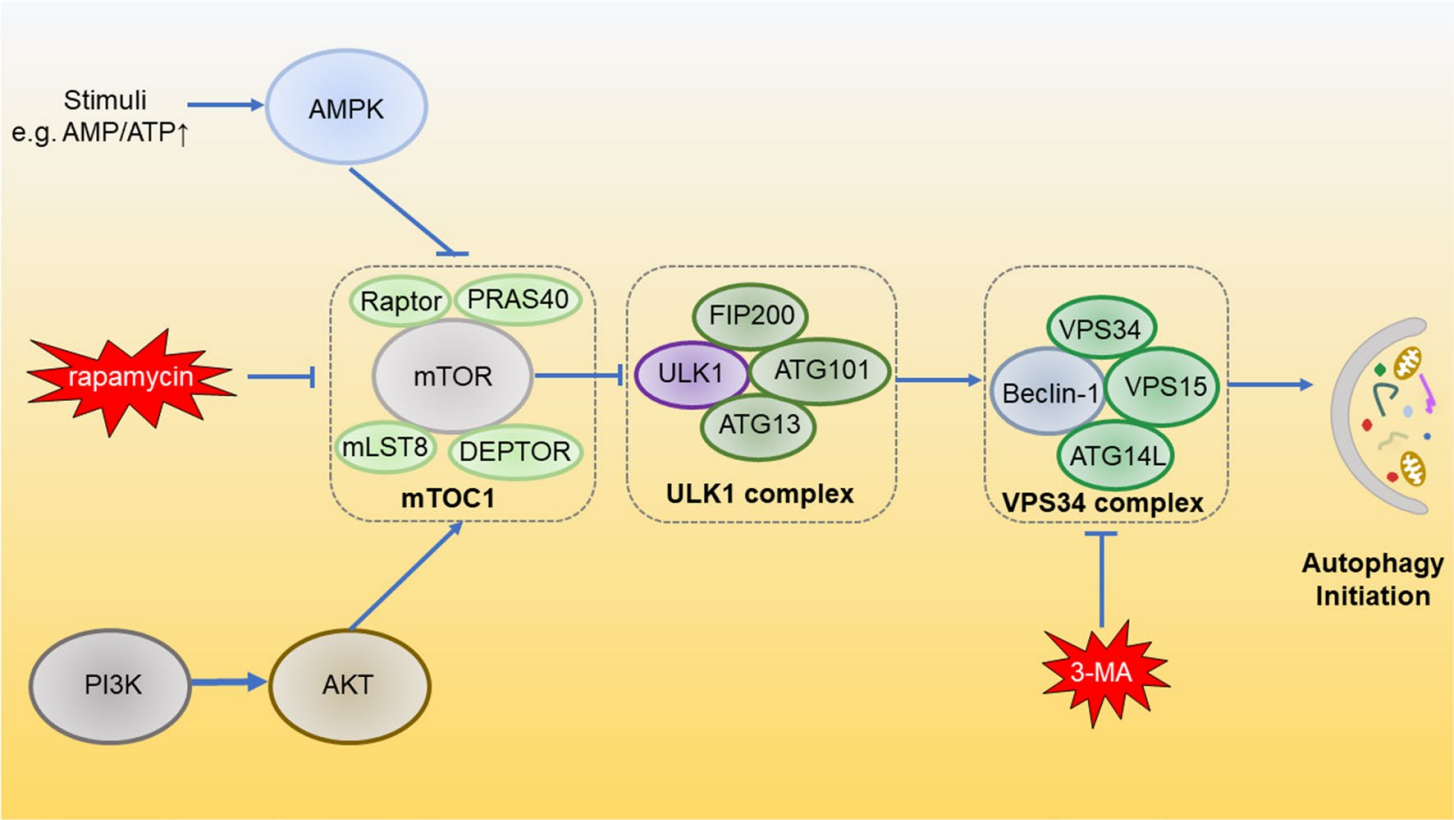
The outline of molecular mechanisms of autophagy initiation. Autophagy is critically regulated by PI3K/AKT/mTOR and AMPK/mTOR signaling cascades. mTOC1 is the conjunction point of the two pathways, which ultimately initiate autophagy through ULK1 complex and VPS34 complex. This process can be inhibited by rapamycin and 3-MA

So far, almost 30 + autophagy-related genes (ATGs) have been identified and their productive proteins ATGs work in the sequential cascade, which have been thoroughly reviewed elsewhere [16]. The molecular mechanisms of autophagic cascades are complicated (Fig. 1). The deprivation of glucose or ATP can upregulate adenosine 5′-monophosphate-activated protein kinase (AMPK). AMPK phosphorylates and deactivates the activity of Mammalian target of rapamycin (mTOR). mTOR has been suggested to play a central role in the initiation of autophagy. mTOR is a major inhibitory modulator of autophagy that suppresses the autophagic cascades upon sufficient energy [10]. In nutrient-rich scenario, the mTOR complex 1 (mTORC1), formed by mTOR, Raptor, PRAS40, mLST8 and DEPTOR, is activated, which subsequently phosphorylates Unc-51-like autophagy-activating kinase-1 (ULK1) at Ser637 and Ser757 and deactivates the ULK1 complex formed by ULK1, ATG13, ATG101, RB1-inducible coiled-coil 1 (RB1CC1, or FIP200) so as to blunt autophagy through the intermediate VPS34 complex (made up of VPS34, VPS15, Beclin-1 and ATG14L) [17]. Due to the deprivation of nutrients, or suppression by mTOR inhibitor rapamycin, mTORC1 is deactivated and autophagy is reversely enhanced [18, 19]. As the upstream pathways of autophagy, the AMPK/mTOR and Phosphoinositide 3-kinase (PI3K)/ protein kinase B (AKT)/mTOR pathways critically regulate autophagy and are responsible for multiple cellular activities and cancer progression.

The confirmation and identification of autophagy can be achieved by several methods. The first one, undoubtedly, is direct observation by electroscope, which can distinguish the different stages. Another one is to detect the expression level of autophagy-associated proteins, such as LC3 and p62. In mammals, LC3, or microtubule-associated protein l light chain 3 (MAP1LC3) is a homologue of Atg8 in yeast [20]. LC3 is subdivided into LC3-I and LC3-II [21]. LC3-I is cytosolic. LC3-II is bound to the membrane of autophagosomes and autolysosomes, moreover, the expression level is positively related to the extent of autophagosome formation. Thus, the increase of LC3-II/LC3-I ratio or LC3-I-to-LC3-II conversion is widely regarded as an autophagy marker in scientific researches [20, 22]. Besides, P62 (Sequestosome 1/SQSTM 1) can be degraded during autophagy, therefore P62 decrease is a remarkable sign indicating autophagy initiation [23]. The autophagic process can be suppressed by several agents, such as chloroquine (CQ), hydroxychloroquine (HCQ) and 3-methyladenine (3-MA). CQ as well as HCQ inhibits lysosomal protease and blocks the fusion between autophagosome and lysosome [24]. 3-MA is the inhibitor of class III PI3K (VPS34) at the upstream of autophagy initiation [17]. Such inhibitors are not purely used for scientific researches, but exhibit strong anti-tumor activities.

## The bipolar roles of autophagy in tumors

**Fig. 2:**
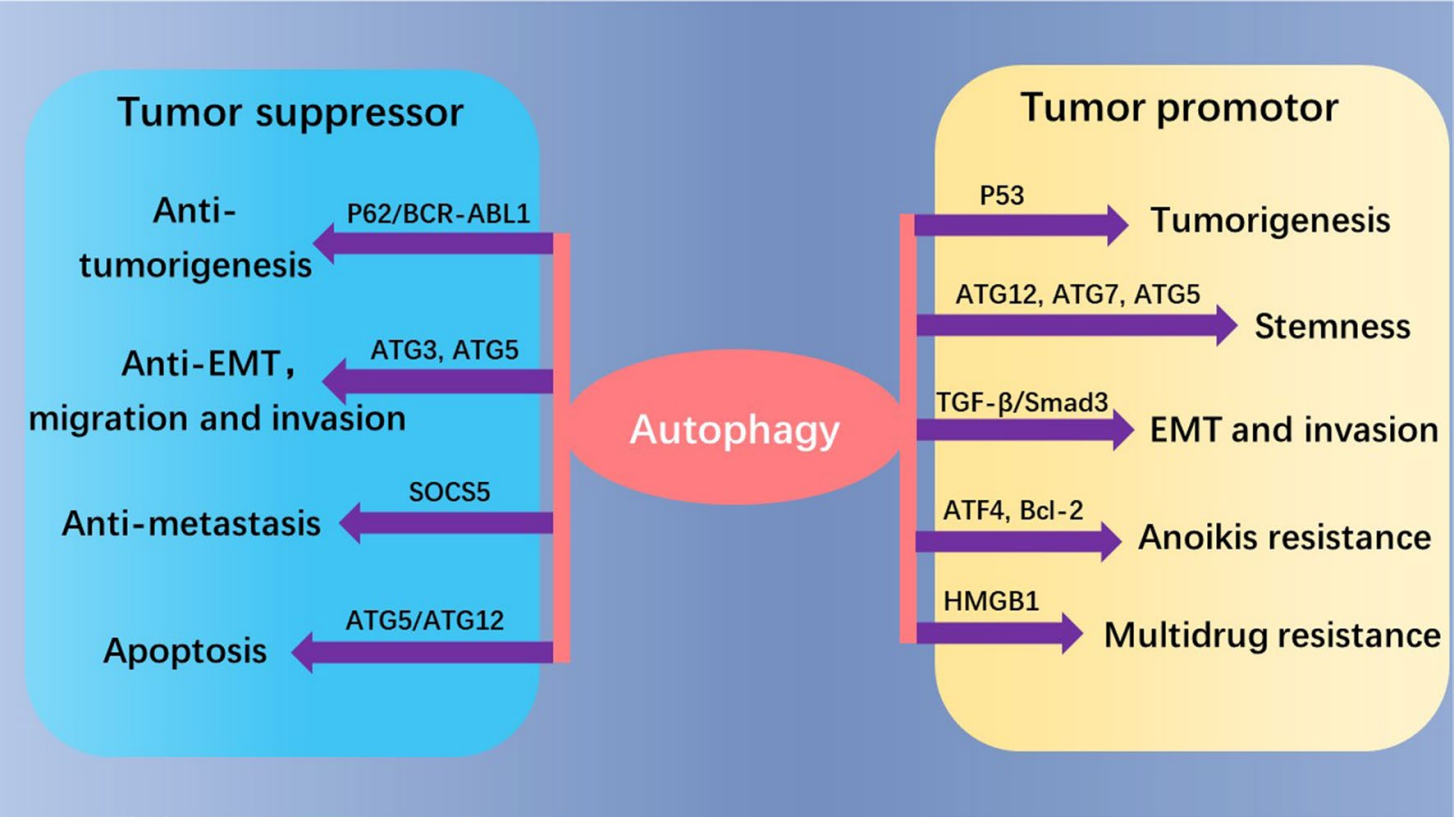
Autophagy either plays tumor suppressive or oncogenic roles in general cancer progression

Dysregulated autophagy is associated with pathogenesis, including skin diseases, pulmonary diseases, neurological diseases and cancers [12, 25–27]. The roles of autophagy in cancers are particularly of note. Autophagy is widely involved in tumor’s biological behaviors including cancer stem cell (CSC) survival [28], cell death [29], distant metastasis [30], multidrug resistance [31] and so on (Fig. 2). The roles of autophagy in cancers are complicated and contradictory, depending on the external environment and nutrient conditions as well as some intrinsic characteristics, including the stages and types of cancer [32] To be more specific, autophagy suppresses tumor progression at early stages by eliminating damaged organelles, misfolded proteins and excessive metabolites, such as reactive oxygen species (ROS). As is known ROS overexpression is harmful for nucleus via oxidative stress that may induce mutagenesis, and autophagy (mitophagy) may sweep away the damaged mitochondria so as to prevent ROS accumulation and tumor development. However, at the late stages or under extreme situations, autophagy provides extra energies as the substances recycle for the tumoral cells and help them adapt to nutrient deprivation or hypoxia conditions, which eventually promotes tumor progression [16, 33, 34].

As a way of keeping homoeostasis, autophagy can eliminate some oncogenic proteins to play a suppressive role, such as P62 [35] and BCR-ABL1 [36]. Take P62 as an example, the accumulation of P62 caused by defective autophagy may lead to ROS production and DNA damages via nuclear factor kappa-B (NF-κB) activation, which may drive tumorigenesis [35]. On the contrary, autophagy can also stimulate suppressor p53 degradation which facilitates tumor growth [37, 38]. Cancer metastasis is complicated and has multiple intersections with autophagy. The main mechanisms underlying metastasis include CSC maintenance, epithelial-mesenchymal transition (EMT) and anoikis resistance. CSC is responsible for cancer metastasis and relapse. Some studies demonstrate that cancer cell stemness of teratocarcinoma and ovary is reduced after knocking down *ATG12*, *ATG7* and *ATG5*, indicating autophagy is a stemness promoter [39, 40]. EMT is a key event for tumor distant metastasis, which renders epithelial cells tendency to invade and migrate. It is demonstrated that autophagy is found to promote EMT and cell invasion via transforming growth factor-β (TGF-β)/Smad3 signaling axis in hepatocellular carcinoma cells HepG2 and BEL7402 [41]. Anoikis is a cell death form after cancer cells detaching from extracellular matrix, thus anoikis resistance is a significant step for metastasis. Yu et al*.*’s study suggests the enhancement of autophagy due to acting transcription factor 4 (ATF4) transcription and B-cell lymphoma-2 (Bcl-2) phosphorylation protects detached prostate cancer cells from anoikis [42]. Although mostly autophagy facilitates metastasis, the opposite result is also reported. Autophagy inhibition by *ATG3* or *ATG5* depletion promotes EMT in several Ras-mutant cancer cells, indicated by the increase of EMT markers like ZEB1, ZEB2 and SNAI2; meanwhile facilitating cell migration and invasion [43]. The inhibition of suppressor of cytokine signaling-5 (SOCS5) protein can augment autophagy by activating PI3K/AKT/mTOR axis but reduce hepatocellular carcinoma metastatic potential [44]. Collectively, the specific role of autophagy in metastasis is uncertain, which requires more investigations.

Although autophagy keeps homeostasis in some extents, it is a means of killing senescent cells, thus it is called the type II programmed cell death. Notably, ATG5 and ATG12 are two significant ones of the regulators. Knockdown of *ATG5* can inhibit autophagy but promote apoptosis. The expression of pro-apoptotic molecules Bax and caspase-3 increase while LC3II/LC3I decreases in ATG5-deficient cells [45]. Moreover, ATG5 cleavage mediated by calpain is able to provoke apoptosis [46]. ATG12 is especially important in mitochondrial apoptosis, in which it associates and inactivates antiapoptotic Bcl-2 and Mcl-1 via the BH3-like region on itself [47]. ATG12 can also conjugate with ATG5. In hepatitis B virus-induced hepatocellular carcinoma, the level of ATG5-ATG12 conjugation is promoted, and ATG12 silencing can moderately increase apoptotic rates of liver cancer cells [48]. It can be seen that they are both potential drug targets for cancer treatments. However, autophagy has cytoprotective roles in anticancer treatment to protect the tumor cells from being killed, such as inducing multidrug resistance. For example, the interplay between autophagy and P-glycoprotein confers resistance against epirubicin in triple negative breast cancer cells [49]. Additionally, as a member of damage-associated molecular pattern (DAMP), the high-mobility group box 1 (HMGB1) has been shown to promote autophagy and induce drug resistance against docetaxel in lung adenocarcinoma and doxorubicin in hepatocellular carcinoma, respectively [50, 51].

## Autophagy participates in TC progression

Even though the role of autophagy is largely unknown in cancer development, there are evidence demonstrating autophagy is involved in multiple events of TC progression, including tumorigenesis, CSC maintenance, cell growth, migration and invasion, cell death as well as therapeutic resistance (Table 1). Holm et al*.* discovered that the inhibition of autophagy via CQ or Lys05 could mitigate CSC and EMT properties, invasion and migration as well as proliferation across PTC cells (MDA-T32, MDA-T68), FTC cells (FTC133) and ATC cells (8505c), thus autophagy is perhaps a tumor promoter [52]. As is mentioned p62 is a key regulator in tumorigenesis, p62 upregulation is examined in PTC tissues and cells. And p62 knockout is a method to inhibit tumor growth, which not only restrains autophagy via the AKT/AMPK/mTOR pathway in TPC-1 cells, but also regulates cell proliferation and apoptosis [53]. On the contrary, Chen et al*.* found p62 reduction was associated with PEST-containing nuclear protein (PCNP) upregulation in TPC-1 and ARO cells, resulting in enhanced autophagy and decreased tumor volume in vivo. Mechanically, PCNP upregulation suppresses Wnt3a upregulation and activation of GSK3β and β-catenin, and the deactivation of Wnt/β-catenin eventually promotes autophagy [54]. Phospholysine phosphohistidine inorganic pyrophosphate phosphatase (LHPP) is a histidine phosphatase with anti-tumor effects that is downregulated in PTC patients’ sample. By introducing LHPP in PTC cell lines including K1, BCPAP and TPC1, LHPP can suppress AKT/mTOR, and activate AMPK signaling to induce cell autophagy and reduce viability [55]. As an upstream molecule of mTOR, AMPK activation inhibits cell growth by suppressing mTOR. OSU-53, an AMPK activator and mTOR inhibitor, can induce autophagy and limit cell proliferation in PTC cells (BCPAP) and ATC cells (Hth-104, Hth-7, SW1736, C643). Interestingly, all these cells carry *BRAF*^V600E^ mutant (BCPAP, Hth-104, SW1736) or *RAS* mutant (Hth-7, C643). On the contrary, the autophagic degree is less obvious in PTC cell TPC1 but absent in FTC133 cells which do not carry *BRAF* or *RAS* mutations. Therefore, gene mutation detection might be required for more precise drug administration in the future [56].

**Table 1:** Agents that mediate cancer progression in TC by regulating autophagy

	Agent	Cells	Molecular axis	Outcome	Effect	Refs.
Autophagy inducer	OSU-53	PTC (BCPAP), ATC (C643, SW1736, HTh-7, Hth-104)	/	Proliferation↓	Inhibition	[56]
	PCNP	PTC (TPC1), ATC (ARO)	PCNP → Wnt/β-catenin → autophagy induction	Apoptosis↑; proliferation↓	Inhibition	[54]
	LHPP	PTC (K1, BCPAP, TPC1)	LHPP → AKT/mTOR and AMPK → autophagy induction	Viability, proliferation, migration↓	Inhibition	[55]
	GX15-070	PTC (BHT101), FTC (ML1, FTC236), ATC (C643, SW1736, Hth7)	/	Cell death; viability↓	Inhibition	[60]
	DAPK2	ATC (TTA1)	DAPK2 → autophagy induction → I-κBα degradation → NF-κB activation	Proliferation↑	Promotion	[64]
	GANT61	ATC (SW1736, KAT-18)	GANT61 → TAK1 → AMPK → autophagy induction	Proliferation↑	Promotion	[66]
Autophagy inhibitor	Lys05 or CQ	PTC (MDA-T32, MDA-T68), FTC (FTC-133), ATC (8505c)	/	EMT, migration and invasion, proliferation↓	Inhibition	[52]
	BIRC7	PTC (BCPAP)	BIRC7 → BECN1 and ATG5 → autophagy suppression	EMT, migration and invasion↑	Promotion	[57]
	CDH6	PTC (BCPAP, TPC1)	CDH6 → (GABAGAP) → autophagy suppression	cell architecture alteration; EMT↑	Promotion	[58]
	LDHA	PTC (BCPAP, TPC1, KTC-1)	LDHA → ATP/ADP ratio → AMPK inhibition → autophagy suppression	EMT, migration and invasion↑,	Promotion	[59]
	BAG3	PTC (KPC1, KPC3), ATC (FRO)	/	Apoptosis↑	Inhibition	[62]
	FOXK2	PTC (BCPAP)	/	Proliferation↑	Promotion	[65]

In BCPAP cells, autophagy can be suppressed by baculoviral IAP repeat-containing 7 (BIRC7) via mTOR, the consequences of which is the downregulation of E-cadherin (epidermal marker) and the upregulation of N-cadherin and Vimentin (mesenchymal markers), which enables further cell migration and invasion [57]. Cadherin-6 (CDH6) is active in EMT and is a marker suggesting high metastatic potential. Similarly, by introducing CDH6 in PTC, autophagy is restrained through the interaction with GABARAP. On the contrary, the silencing of CDH6 activates autophagy and partially reverses EMT [58]. Moreover, lactate dehydrogenase A (LDHA) overexpression can reduce the ADP/ATP ratio and activate AMPK/mTOR pathway, then inhibit cell autophagy and promote EMT, migration and invasion. The cotreatment of LDHA inhibitor (FX11) and HCQ further suppress tumor growth more than the use alone, indicating a protective role of autophagy [59]. These results suggest the anti-metastasis property of autophagy in some PTC cells.

As a way of cell death, autophagy has multiple links with apoptosis. GX15-070 (Obatoclax) is mimetic of BH3 that targets anti-apoptotic protein Bcl-2 and restrains cell viability as mentioned above. GX15-070 administration triggers cell death by means of a mixture of autophagic cell death, apoptosis and necrosis in multiple FTC, PTC and ATC cell lines to restrain cell viability [60]. The silencing of transmembrane protein (TMP) 21 in TPC1 cells can reduce cell viability and enhance apoptotic rates. Due to the lack of TMP21, the levels of P-mTOR and P-S6K are decreased, and the P-AMPK and ratio LC3II/LC3I increased, suggesting autophagy may play a role in inducing apoptosis [61]. Autophagy in nutrition deficiency is usually a pro-survival mechanism to bear extreme condition. In starvation environment treated by EBSS, autophagy attenuation is led by ectopic Bcl-2-associated athanogene 3 (BAG3) expression, which subsequently elicits apoptosis in FRO cells [62]. Similarly, the inhibition of autophagy by ATG7 siRNA enhances tumor necrosis factor-related apoptosis-inducing ligand (TRAIL)-induced apoptosis in FRO cells, which implies a prosurvival role. On the contrary, such results are not replicated in TPC-1 cells. However, in TPC-1 cells, the autophagy inhibition reduces apoptosis in response to TRAIL, indicating the proapoptotic role of autophagy [63]. Death-associated protein kinase 2 (DAPK2) is a modulator for TRAIL-induced apoptosis. An additional study in another ATC cell line TTA1 shows that autophagy can be enhanced by DAPK2, consequently degrades inhibitory-κBα (I-κBα) and activates NF-κB signaling, which renders the aggressive potential [64]. Recently, autophagy promotion is witnessed in forkhead box class K 2 (FOXK2)-silencing BCPAP and BHT-101 cells, resulting in constrained cell proliferation and suggesting a suppressive role [65]. Autophagy inhibition by sonic hedgehog (Shh)-associated TGF-β-activated kinase (TAK1) siRNA has been demonstrated to potentiate GANT61 (a Shh pathway inhibitor)-induced apoptotic and antiproliferative effects, reflecting a cytoprotective role of autophagy in SW1736 ATC cells [66]. Autophagy suppression and apoptosis promotion are also observed in Wilms’ tumor 1 (WT1)-knockdown IHH4 and BCPAP cells [67]. Conversely, ASAP1 (ArfGAP with SH3 Domain, Ankyrin Repeat and PH Domain1) knockdown in PTC MDA-T32 and MDA-T85 cells triggers formation of autophagosomes and promotes autophagy through the mTOR pathway. As ASAP1 is upregulated in PTC tissue, this kind of autophagy is proposed to be inhibitory [68].

## Autophagy regulates metabolism of TC

Cancers have their metabolism altered to meet the high energy needs for uncontrolled cell growth. For example, they are able to catabolize fructose, arginine and acetate for energy production [69–71]. In addition, because the speed of angiogenesis could not catch up with the rate of rapid tumor growth, thus cancer cells have to overcome the influence of hypoxia or shortage of nutrients. These alterations are closely correlated to multiple tumoral cellular events, so they have potential to be druggable targets for clinical treatments.

Autophagy interacts with TC metabolism through multiple pathways. Glucose deprivation is an inducer of autophagy in FTC133 and WRO cells, which also impairs cell proliferation and migration but induces apoptotic cell death. Moreover, the glucose uptake is negatively associated with phosphatase and tensin homolog deleted on chromosome 10 (PTEN) and p53 level, which are frequently mutated or lost in TC. Therefore, TC carrying such mutations may have increased glucose uptake and dysregulated autophagy [72]. Glucose metabolism in cancer cells is preferentially mediated by aerobic glycolysis for energy production regardless of the presence of oxygen, known as the Warburg effect [73]. Sirtuin6 (SIRT6) is a type of histone deacetylases that specifically induces histone H3 lysine 56 acetylation (H3K56ac) and H3 lysine 59 acetylation (H3K9ac). In TPC-1 and K1 cells, SIRT6 overexpression leads to ER stress by elevating ROS production and then activates autophagy. Subsequently, the SIRT6-induced autophagy is able to degrade the Warburg effect-related protein glucose transporter 1 (GLUT1) and inhibit this process with glucose uptake downregulation. Reversely, the introduction of autophagy inhibitor or ROS inhibitor would promote tumor growth in vivo [74]. Glutaminolysis is a hallmark of cancer metabolism. The glutaminase can convert glutamine to glutamate and NH3 for fuel [75]. A study has revealed that the metabolism of PTC cell lines (K1, IHH4, BCPAP, and TPC-1) is glutamine-dependent. The inhibition of glutaminase suppresses glutaminolysis, and simultaneously decreases cell proliferation, migration and invasion. Moreover, the inhibition also suppresses the phosphorylation of mTOR, p70s6k, 4EBP1 and ULK1, which induces autophagy and apoptosis [76]. These studies demonstrate autophagy enhancement may alter and block nutrition supply, which can be used as drug target for cancer therapy.

## Autophagy can be regulated by radiotherapy in TC

Radiotherapy including internal and external radiotherapy (ERT) is both significant for whole period TC management. It is known that thyrocytes are characterized by iodine uptake ability, thus the uptake of radioactive iodide which destroy the surrounding remnant thyroid tissue is used for postoperative treatment, known as RAI therapy or internal radiotherapy [77]. ERT is also a crucial adjuvant therapy in TC management, especially for ATC patients that almost all the patients demand it. ERT also has great therapeutic effects for high-risk DTC patients after surgeries and RAI, indicated by the high locoregional recurrence-free survival [78].

### ERT may promote autophagy in squamous TC cells

Autophagy and apoptosis could be induced by radiation, whereas autophagy may decrease the p53-dependent apoptosis. By constraining autophagy via 3-MA or siRNA Beclin-1 in SW579 (squamous cell carcinoma) cells, one study has shown that the degree of apoptosis is conversely enhanced, suggesting a cytoprotective role of autophagy [79]. Their subsequent research reveals that X-ray radiation facilitates ROS generation that may enhance autophagy. And the application of ROS inhibitors like superoxide dismutase can reactivate cell apoptosis by blocking autophagy [80]. These results point out that the combination of ERT and autophagy inhibitors probably result in better anti-tumor effects, at least in squamous cell carcinoma of thyroid.

### Iodide accumulation elevates autophagy and suppresses TC

Digoxin exposure has been noticed to promote autophagy and accelerate iodine accumulation in mice model bearing TC. The tumor volume is shrank after the treatment, thus it is assumed that autophagy is positively correlated to iodide uptake that constrains tumor growth [81]. This result is also confirmed in other studies. Undergoing cell transfection with small activating RNA, the SW579 expresses upregulated sodium/iodine symporter (NIS) on its surface, and concomitantly the autophagy is enhanced. In turn, treating with rapamycin and 3-MA respectively increases or decreases NIS levels, indicating a regulatory loop between autophagy and NIS. NIS enhancement elevates autophagy activity and iodine uptake as well as constrains cell viability through AMPK/mTOR pathway [82]. Moreover, the positive regulation of NIS by autophagy has been detected in non-medullary thyroid carcinoma samples. Autophagy activation is found to prompt RAI uptake and a promising predictor to evaluate tumor remission rates [83]. The NIS can also be regulated by HMGB1. HMGB1 is highly expressed in clinical samples and FTC-133 as well as TPC-1 cells, and it may induce autophagy which, oppositely, accelerates NIS degradation, thereby decreases iodine uptake and disrupts RAI therapy [83]. Notably, the translocation of HMGB1 from nucleus to cytosol and ROS generation are two significant processes in the study, which may explain the dual roles of autophagy on NIS. And the different cell properties may also lead to the opposite results, but the specific mechanisms still remain to be elucidated.

## Autophagy is a promising target for TC treatments

### Small molecules

Reversine is a man-made small molecule with dedifferentiation activity. By treating FTC cells, this agent suppresses AKT/mTOR/p70S6K pathway to induce autophagy and decrease cell viability [84]. Doxorubicin and radiation can respectively induce autophagy in 8505-C and TPC-1 cells. On the contrary, inhibition of autophagy suppresses chemosensitivity and radiosensitivity, indicating autophagy is tumor-suppressive [85]. Their subsequent research demonstrated that the combination of doxorubicin or radiation with RAD001 (everolimus, an analogue of rapamycin and oral mTOR inhibitor) can augment autophagy and therapeutic efficacies more than the single treatment, and Met was identified as the essential mediator in the process [86]. The addition of RAD001 can further potentiate effects of tyrosine kinase inhibitors like sunitinib and sorafenib to activate autophagic afflux and inhibit proliferation in MTC [87]. In metastatic TC cells, autophagy can be raised by valproic acid, inducing growth inhibition and apoptosis [88]. These observations collectively indicate that autophagy promotion is useful for TC treatment.

### Targeted therapy

The conventional treatment of TC like surgeries and RAI therapies can be used for most of the early or metastasized cases. For less differentiated TC or MTC and advanced TC, targeted therapy is of significance, in which cabozantinib, sorafenib and lenvatinib have been approved for clinical uses. The common drug targets include fibroblast growth factor receptor (FGFR), vascular endothelial growth factor receptor (VEGFR), RET, BRAF, etc.

Apatinib has been approved for advanced or metastatic gastric cancer in China, which selectively targets VEGFR-2 to suppress angiogenesis [89]. Recently, apatinib has shown benefits in patients with locally advanced or metastatic, RAI–refractory differentiated TC indicated by longer progression-free survival and overall survival [90]. In ATC cell lines KHM-5 M and C643, apatinib treatment elevates the formation of autophagosome and apoptosis by regulating the AKT/mTOR signaling pathway. However, the co-treatment of apatinib and CQ ameliorates autophagy while further increases apoptosis in such cell lines, indicating a protective role of autophagy [91]. Similar observations are also reported in PTC. Apatinib-treated cells exhibits autophagy, and the inhibition of which by HCQ accelerates cell apoptosis and decrease tumor growth in vivo [92]. These results suggest apatinib may combine with anti-autophagy drugs to better exert anti-tumor function.

Vemurafenib is a highly selective BRAF inhibitor (mainly the V600E mutation) used for unresectable melanoma [93]. As BRAF^V600E^ mutant is estimated in 60% of PTC cases, it might be a promising drug for refractory PTC, which has been already indicated in some clinical trials [94–96]. Vemurafenib induces autophagy via activating endoplasmic reticulum (ER) stress response mediated by the elevation of eIF2a phosphorylation and CHOP expression in BRAF-mutant cells. Nevertheless, the vemurafenib-induced autophagy plays a protective role against cell death, and the inhibition of autophagy by either HCQ or ATG5 siRNA treatment enhances vemurafenib efficacy [97]. Treatment with PLX4720, the progenitor of vemurafenib, could activate AMPK signaling by phosphorylating the residue Thr172 and then phosphorylate ULK1 to induce autophagy in BHT101 ATC cells. The simultaneous treatment with BRAF^V600E^ inhibitor and autophagy inhibitor (CQ) leads to increased apoptosis [98]. With the application of vemurafenib growing, some studies report the drug resistance, which limits its clinical prospects. Run et al*.* noticed that HMGB1 was perhaps one of the inducers that conferred cell resistance to vemurafenib. Upon vemurafenib exposure in BCPAP cells, knockdown of HMGB1 decreased autophagy but reversed the sensitivity to vemurafenib [98]. In K-1 and BCPAP cells, introduction of redox factor-1 (Ref-1) inhibitor E3330 can strikingly induce autophagy and senescence phenotype towards vemurafenib treatment, and strengthen drug sensitivity [99].

From these results, it can be concluded that the targeted therapies can inevitably induce autophagy in thyroid cancer treatment and it is a cytoprotective factor in most cases, therefore the co-inhibition of autophagy can further potentiate these targeted drugs’ effects.

### Natural products

The potential anti-autophagy and anti-tumor roles of natural bioactive agents have gained a lot of attention recently. *Phellinus linteus* polysaccharide (PLP) is the main bioactive compound of *Phellinus linteus* with anti-tumor roles. PLP was found to induce not only macroautophagy (autophagy), but also mitochondrial autophagy and ER-autophagy in TPC-1 and SW579 cells. Together with apoptosis, these events collectively ameliorate the malignant potentials [100]. Apigenin is flavonoid with anti-inflammatory, antioxidant and anticancer functions. By treating BCPAP with apigenin, autophagy is induced through the p62/Keap1/Nrf2 pathway and the production of ROS, promoting autophagic cell death [101]. Flavokawain B also induces autophagy in TC lines by deactivating mTOR, and meanwhile phosphorylating AMPK. However, on this occasion, autophagy plays a cytoprotective role that the cell lines show more significantly reduced cell viability cotreated by Flavokawain B and autophagy inhibitor CQ or 3-MA [102].Punicalagin is a kind of tannins. In BCPAP cells, it induces autophagic cell death rather than apoptosis. Concomitantly, the level of phosphorylated ERK1/2 and p38 are upregulated whereas phosphorylated p70S6 and 4E-BP1 are downregulated, indicating MAPK pathway activation and mTOR inhibition during the process [103]. Curcumin treatment can result in autophagic cell death in TC cell lines by activating canonical MAPK pathway but inhibiting AKT/mTOR/p70S6K pathway [104]. Honokiol also induces autophagy in the ARO, WRO and SW579 cells via AKT/mTOR and MAPK pathways [105]. Similarly, both allicin and mulberry anthocyanin induce autophagic cell death in SW1736 and HTh‑7 cells by suppressing the AKT/mTOR/S6 pathway [106, 107]. Capsaicin triggers calcium influx in ATC cells via transient receptor potential vanilloid-1 (TRVP1), and the overload of calcium suppresses AKT pathway and induces autophagy. Furthermore, capsaicin is involved in the autophagic degradation of OCT4A, a cell stemness regulator. The reduction of stemness can probably ameliorate ATC aggressiveness [108]. In a word, these agents have great pharmacological and economic benefits, and more translational researches and clinical trials are required in the future.

### Clinical trials

At the time of writing, unfortunately, the results of clinical trials on TC by inhibiting autophagy are disappointing. In fact, CQ and HCQ have been used as autophagy inhibitors and investigated elsewhere in clinical trials of breast cancer and metastatic pancreatic cancer patients [109, 110]. Moreover, the sole use of CQ or HCQ could only lead to limited effects, while combination of these inhibitors with chemotherapeutic drugs can reverse the outcomes. For instance, the phase II study of HCQ monotherapy showed negligible pharmacological effects in metastatic pancreatic adenocarcinoma patients, whereas the combination of nab-paclitaxel and gemcitabine plus HCQ can preoperatively improve the Evans grade histologic response and facilitate immune infiltration in resectable pancreatic adenocarcinoma [111]. Even though there is no direct evidence proving the autophagy inhibitors work in TC patients, their therapeutic efficacy has been preliminarily uncovered in cell lines and mice model [91]*.* How does the combination therapy work in patients with TC still remains to be seen.

## Concluding remarks and future perspectives

**Fig. 3:**
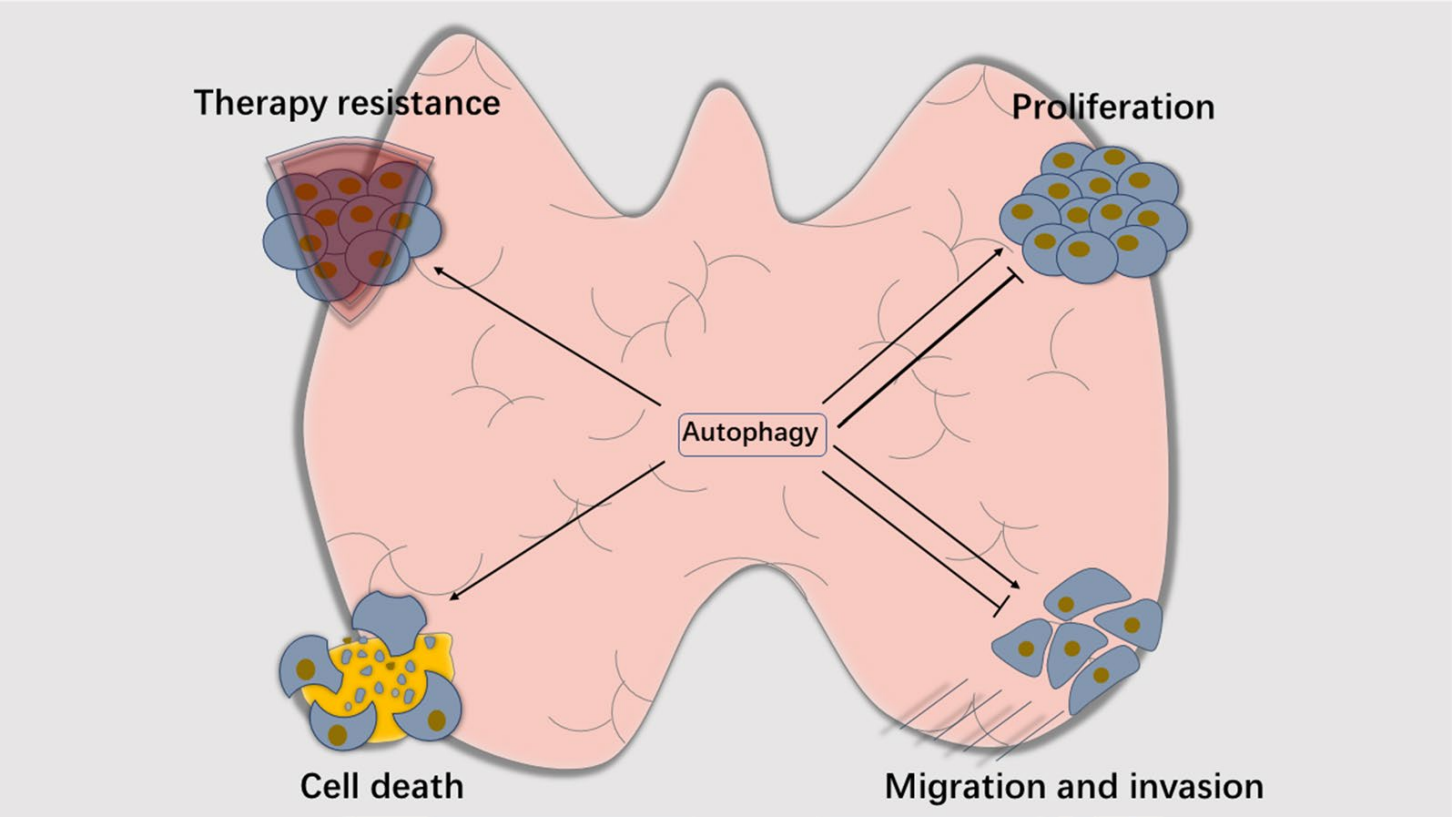
Autophagy influences progression and development in TC

Autophagy is a dual-sword in the process of tumorigenesis. On the one hand, it suppresses tumor cell growth by degrading oncogenic proteins or inducing autophagic cell death. On the other hand, autophagy preserves energies when bearing miscellaneous stress or stimuli which promotes cell survival. By modulating a variety of molecules and pathways, autophagy plays a dispensable role in TC progression (Fig. 3). It is well believed that autophagic cascades could be potential druggable targets for cancer therapy including TC, for instance, the lysosome inhibitors, ATG4 inhibitors and VPS34 inhibitors [25].

The fundamental question—whether autophagy is tumor suppressive or oncogenic in TC—is still arguable. Autophagy enhancement has been reported to exhibit dual roles as previously discussed. Recently, Kazakova et al*.* evaluated the role of autophagy inducers, namely, cisplatin, rapamycin, irradiation and sorafenib in three cell lines TPC1 (PTC), ACT1 (ATC) and KTC1 (poorly-differentiated TC). Unexpectedly, compared with the CQ-cotreated group, they all play a pro-survival role in TC cells, indicated by lower apoptotic rates [112]. The reasons causing the discrepancy could be various, such as different cell models, insufficient analyses or assessments, data misinterpretation and improper group settings. And the culture media with or without sufficient nutrition may also contribute to variation. It is noticed that the roles of autophagy in targeted drugs-treated TC cells and natural agents-treated cells are seemingly contradictory as previously discussed, and the different treatment is to use autophagy inhibitors or not. Therefore, it is assumed that autophagy is actually playing a pro-survival role in both treated groups, but it is covered in the natural agents-treated cells due to the lack of autophagy inhibitors. The study on Flavokawain B further confirms the inference but more work is still required in the future [102]. Therefore, identifying the balance point that maximizes the effects of autophagic cell death and meanwhile minimizes the malignant potentials and therapeutic resistance could be challenging, which requires more investigations.

In this review, we mainly focus on how autophagy specifically modulates TC progression and what measures can be applied to restrain cancer development. Although the investigation towards TC and autophagy has made great progress, quite a lot of issues still are unsettled, in particular, the translational contexts for clinical benefits. In-depth researches are demanded in the future work for the management of TC on the basis of autophagy.

## Data Availability

Not applicable.

## Abbreviations

TC: Thyroid cancer
PTC: Papillary thyroid cancer
FTC: Follicular thyroid cancer
ATC: Anaplastic thyroid cancer
RAI: Radioactive iodine
ATGs: Autophagy-related genes
AMPK: Adenosine 5′-monophosphate-activated protein kinase
mTOR: Mammalian target of rapamycin
ULK1: Unc-51-like autophagy-activating kinase-1
RB1CC1: RB1-inducible coiled-coil 1
PI3K: Phosphoinositide 3-kinase
AKT: Protein kinase B
MAP1LC3: Microtubule-associated protein l light chain 3
P62: Sequestosome 1
CQ: Chloroquine
HCQ: Hydrochloroquine
3-MA: 3-Methyladenine
CSC: Cancer stem cell
ROS: Reactive oxygen species
NF-κB: Nuclear factor kappa-B
EMT: Epithelial–mesenchymal transition
TGF-β: Transforming growth factor-β
ATF4: Acting transcription factor 4
Bcl-2: B-cell lymphoma-2
DAMP: Damage-associated molecular pattern
HMGB1: High-mobility group box 1
PCNP: PEST-containing nuclear protein
LHPP: Phospholysine phosphohistidine inorganic pyrophosphate phosphatase
BIRC7: Baculoviral IAP repeat-containing 7
CDH6: Cadherin-6
LDHA: Lactate dehydrogenase A
TMP: Transmembrane protein
BAG3: Bcl-2-associated athanogene 3
TRAIL: Tumor necrosis factor-related apoptosis-inducing ligand
DAPK2: Death-associated protein kinase 2
I-κBα: Inhibitory-κBα
FOXK2: Forkhead box class K 2
Shh: Sonic hedgehog
TAK1: TGF-β-activated kinase
WT1: Wilms’ tumor 1
ASAP1: ArfGAP with SH3 Domain, Ankyrin Repeat and PH Domain1
PTEN: Phosphatase and tensin homolog deleted on chromosome 10
SIRT6: Sirtuin6
H3K56ac: Histone H3 lysine 56 acetylation
H3K9ac: H3 lysine 59 acetylation
GLUT1: Glucose transporter 1
NIS: Sodium/iodine symporter
VEGFR-2: Vascular endothelial growth factor receptor-2
Ref-1: Redox factor-1
PLP: Phellinus linteus polysaccharide
TRVP1: Transient receptor potential vanilloid-1
